# Study on the Feasibility of Self-Assembling Peptides as a Three-Dimensional Culture Tool for Drug Screening of Colorectal Adenocarcinoma Cells

**DOI:** 10.3390/gels11060394

**Published:** 2025-05-27

**Authors:** Yu Gao, Di Su, Jiawei Zhao, Zhongli Luo, Xuemei Lin

**Affiliations:** 1 College of Basic Medical Science, Chongqing Medical University, Chongqing 400016, China; 18875208117@163.com; 2 Molecular Medicine and Cancer Research Center, College of Basic Medical Science, Chongqing Medical University, Chongqing 400016, China; sd199877@163.com (D.S.); zjw980312@163.com (J.Z.); zhongliluo@163.com (Z.L.)

**Keywords:** self-assembled peptides, colorectal adenocarcinoma cells, three-dimensional culture, drug screening

## Abstract

In recent years, the incidence of colorectal cancer has been increasing annually. During the research on tumor treatment strategies, the translation of fundamental research findings into clinical applications has often been constrained by the limitations of existing tumor models, with few breakthroughs achieved to date. Therefore, our objective is to explore the feasibility of utilizing self-assembling short peptides (SAPs) to construct a three-dimensional (3D) culture system for colorectal adenocarcinoma cells for anticancer drug screening. By characterizing the physicochemical properties and biocompatibility of SAP SCIOBIO III, we demonstrated that it can rapidly self-assemble into a nanofibrous scaffold under ionic triggers, supporting 3D cell culture. Subsequently, anticancer drug sensitivity tests were conducted on both 2D and 3D culture systems of colorectal adenocarcinoma cells. The results indicate that SCIOBIO III can mimic the extracellular matrix and serves as an ideal scaffold for constructing a 3D cell culture microenvironment. We successfully established a 3D culture model for colorectal adenocarcinoma cells and effectively screened anticancer drugs, which holds promise for advancing the development of personalized anticancer drug screening technologies.

## 1. Introduction

Colorectal cancer is one of the most prevalent and deadly cancers worldwide, primarily due to metastasis and drug resistance [1]. For a long time, cellular and animal models have been widely used in the study of various human diseases [2]. Among these, cell models are among the most commonly applied research tools [3]. In the past, research models for colorectal cancer primarily consisted of tumor cell lines and cell line xenografts [4]. These models have many limitations. Firstly, they lack the microenvironment and heterogeneity present in clinical tumors. Secondly, they struggle to accurately reflect the pathogenesis of colorectal cancer in clinical settings. Finally, they have difficulty precisely expressing drug responses in colorectal cancer [5].

In recent years, the emergence of 3D in vitro culture technology has enabled the establishment of 3D in vitro culture systems for tumor cells [6]. This system is capable of conducting extensive drug screening and is of significant value in evaluating the efficacy and safety of drugs [7]. Tumor cell 3D culture can simulate their microenvironment, retain interactions between tumor cells, reflect somatic mutations in tumors, and demonstrate the biological characteristics of tumors [7,8]. The unique regional microenvironment surrounding tumor cells profoundly influences their biological properties once the tumor reaches a detectable size [9]. However, these properties cannot be replicated in two-dimensional (2D) culture systems [10]. Therefore, the ideal model for studying tumor diseases is the 3D culture of tumor cells [11]. However, most contemporary methods for 3D tumor cell culture employ matrices derived from animals. These matrices include various murine-derived growth factors that could introduce misleading and physiologically irrelevant pleiotropic effects on 3D tumor cell culture models, such as type IV collagen [12,13].

Hydrogels are a novel type of biomaterial with multiple excellent properties, offering broad application prospects. They exhibit good permeability, degradability, and biocompatibility [14,15]. Due to their biomimetic matrix characteristics, hydrogels offer a porous microenvironment with high water content, which is conducive to cell infiltration [16,17]. Research has indicated that due to the high water content and strong permeability of the nano/micro-porous structure of hydrogels, they can effectively encapsulate cells without impeding material exchange between them. Consequently, hydrogel matrices can be utilized for the in vitro cultivation and growth of various cells [18,19]. Molecular self-assembly is a natural process where ordered structures spontaneously form in biological systems. In recent years, various designed amino acid sequences [20] have been developed using the concept of self-assembly. Well-designed peptides can mimic the 3D structure of extracellular matrices in vivo, and since short peptides ultimately break down into amino acid monomers after metabolism, this characteristic avoids the risk of contamination by animal-derived pathogens [21,22]. SAP can self-assemble into a nanofiber mesh scaffold (with a diameter of 10 nm and pore sizes ranging from 5 to 200 nm) in a metal cationic environment [23]. SAP can form stable β-structured frameworks, which under certain conditions assemble into 3D mesh structures. The porosity of these structures is similar to that which promotes the proliferation, differentiation, and adhesion of various cells in the microenvironment of the body [24]. SAP are functional polymers synthesized from natural amino acids, which are considered nanomaterials [25]. Under certain conditions, SAP can autonomously form hydrogels with a nano-fiber network structure. For instance, RADA16 and KLD16 can form highly hydrated, permeable hydrogels when stimulated by metal salt ions [18,26]. Currently, SAP has been widely applied in 3D cell culture, building colorectal adenocarcinoma cell models in a 3D environment based on in vitro 3D cell culture technology, which helps maintain tumor heterogeneity and retain the complex influence of the surrounding microenvironment [27].

At present, cytotoxic chemotherapy remains the dominant approach in the clinical treatment of colorectal cancer. The 3D tumor cell culture model, one of the most promising drug screening models in precision medicine, holds great potential in providing rapid, precise, and objective dosing regimens for clinicians treating colorectal cancer [28].

In this study, we investigated the high-purity (>95%) D-type self-assembled peptide SCIOBIO III. We observed the peptide’s ability to form hydrogels at both microscopic and macroscopic levels. Utilizing this short peptide as an in vitro culture matrix, we grew 3D cultures of the colorectal adenocarcinoma cell line CACO-2 (ATCC, American Type Culture Collection) and analyzed its growth activity within the hydrogel. Finally, we tested the drug sensitivity of the 3D culture model constructed with colorectal adenocarcinoma cells CACO-2, exploring the feasibility of employing the 3D culture system of CACO-2 cells as a platform for screening anticancer chemotherapy drugs.

## 2. Results and Discussion

### 2.1. Characterization of the Physicochemical Properties of SCIOBIO III

#### 2.1.1. AFM Detection of the Microstructure of SCIOBIO III

**Figure 1:**
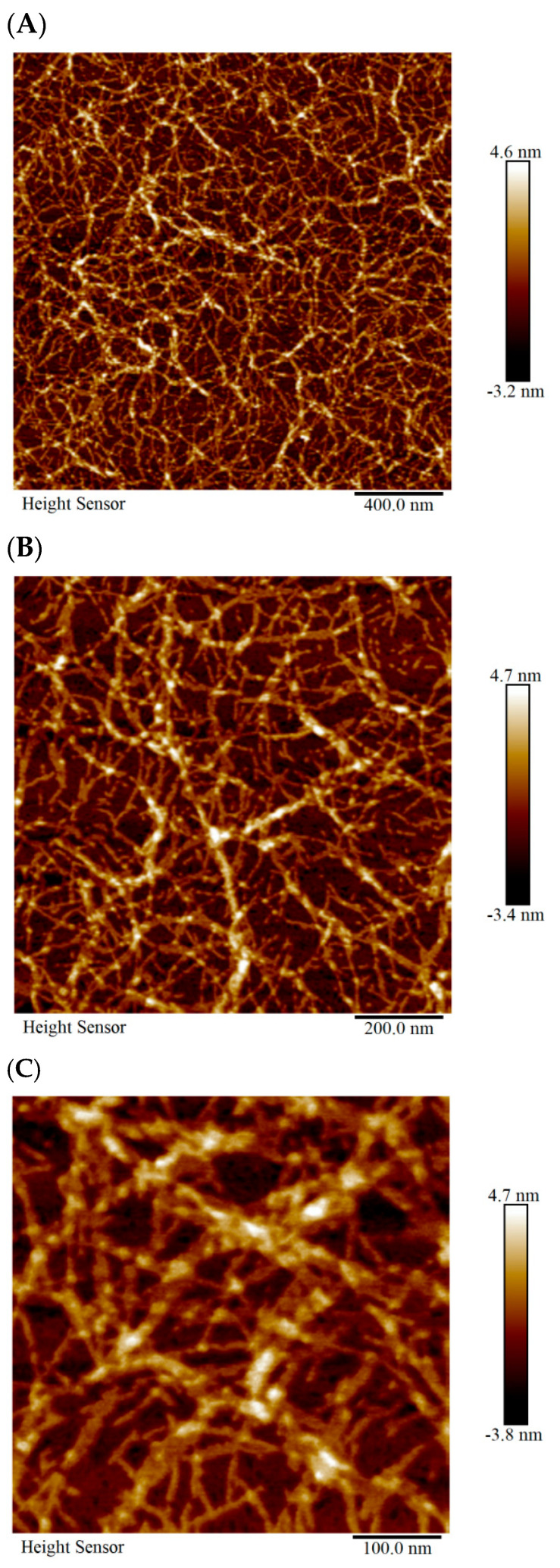
Microstructural analysis of SCIOBIO III. (**A**) AFM result of SCIOBIO III at 400 nm. (**B**) AFM result of SCIOBIO III at 200 nm. (**C**) AFM result of SCIOBIO III at 100 nm.

Atomic force microscopy reveals that SCIOBIO III peptide molecules can form complex nano-network-like structures during self-assembly. Regularly arranged dense nanofibers, approximately 100 nm in length and 20 nm in width, are observed interweaving to construct a 3D nano-network scaffold system (Figure 1).

#### 2.1.2. Cryogenic-Scanning Electron Microscope (Cryo-SEM) Detection of the Microstructure of SCIOBIO III

**Figure 2:**
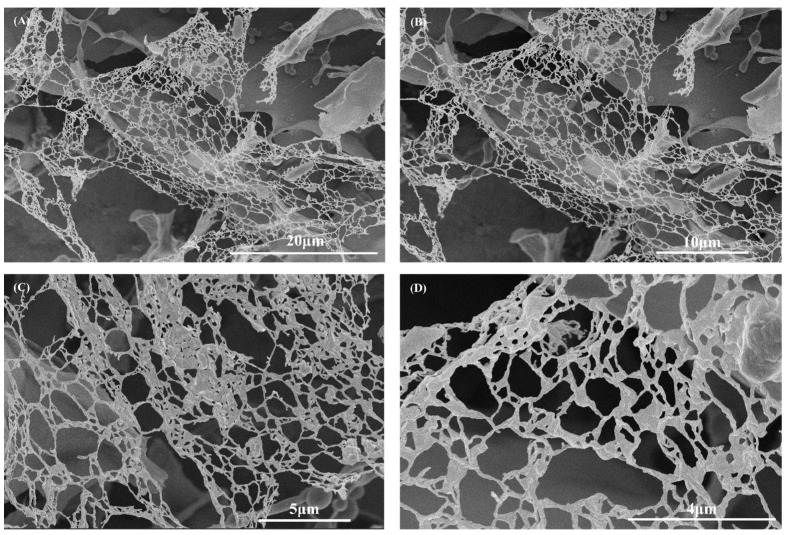
Microscopic structural characterization of SCIOBIO III under Cryo-SEM. Scale bar: (**A**) 20 µm, (**B**) 10 µm, (**C**) 5 µm, (**D**) 4 µm. Imaging parameters: high voltage (HV): 5.00 kV; work distance (WD): 4.00 mm; magnification (MAG): (**A**) 8000×, (**B**) 10,000×, (**C**) 20,000×, (**D**) 40,000×.

The results from biological Cryo-SEM indicate that SCIOBIO III short peptides can form an ordered nano-net fiber scaffold structure. Cryo-scanning at low temperatures reveals that after self-assembly, the short peptides form 3D nanofiber network-like structures with a uniform diameter (nanotube diameter range: 7–24 nm). These fibrous structures, through mutual cross-linking, constitute a highly ordered porous scaffold system (Figure 2).

#### 2.1.3. Congo Red/Aniline Blue Detection of the Macroscopic Morphology of SCIOBIO III

**Figure 3:**
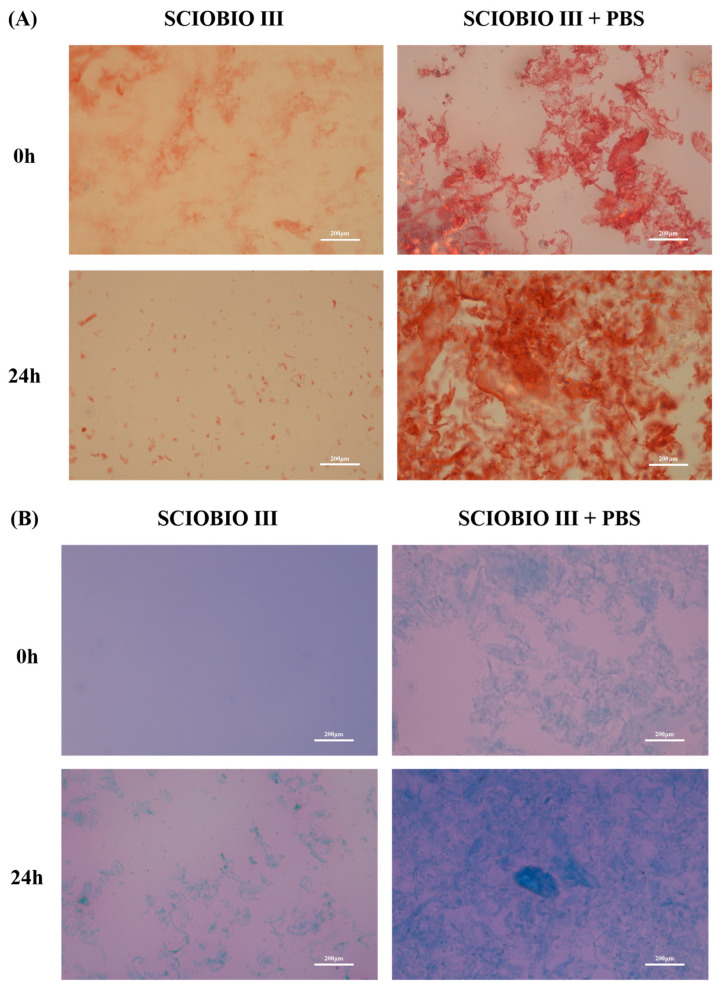
Congo red/aniline blue double staining results. (**A**) Congo red staining of SCIOBIO III short peptides at 0 h and 24 h; (**B**) aniline blue staining of SCIOBIO III short peptides at 0 h and 24 h. Scale bar: 200 µm.

The macroscopic morphology of SCIOBIO III short peptides was analyzed using Congo red and aniline blue staining. The staining results revealed that the short peptide solution mixed with PBS began forming fine, flaky film structures at 0 h. After 24 h at room temperature, the self-assembled short peptides exhibited a dense film-like morphology with a stable lamellar structure distribution. In contrast, the short peptide solution mixed with sterile distilled water showed sparse and uneven, flowing sand-like macroscopic structures at both 0 h and after 24 h of incubation. Thus, this study investigates the rapid self-assembly of SCIOBIO III short peptides under ionic stimulation to form stable fibrous scaffolds. This finding lays the foundation for constructing 3D tumor cell culture systems (Figure 3).

### 2.2. SCIOBIO III for 3D Culture of Colorectal Adenocarcinoma Cells

#### 2.2.1. Three-Dimensional Culture of CACO-2 in SCIOBIO III

**Figure 4:**
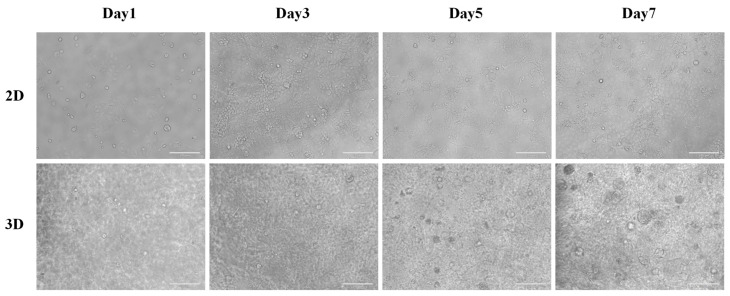
The morphology of CACO-2 in 2D and 3D culture systems on day 1–7 was examined under optical microscope. Scale bar: 200 µm.

Observations of cell proliferation dynamics and image recording were conducted at 24 h, 72 h, 120 h, and the first week after the establishment of 2D and 3D culture systems in both experimental and control groups. Under conventional 2D culture conditions, CACO-2 cells formed an island-like aggregation pattern, with single-layer cells adhering to the surface and proliferating outward. The cell morphology was irregular and varied in size, with some large cells containing vacuoles. When cell culture exceeded three days, the number of cells significantly increased, and density gradually rose. By day five, the microscope field was already completely occupied by proliferating cells. Meanwhile, CACO-2 cells grown in SCIOBIO III short peptide matrices displayed a spherical morphology, with a spatially 3D structure. They exhibited good proliferative activity within the 3D mesh scaffold constructed from peptides, showing significant increases in cell numbers and volume after 72 h of culture. These figures confirm that SCIOBIO III peptide materials successfully mimic the in vivo microenvironment, providing suitable 3D culture conditions for colorectal adenocarcinoma cell lines (Figure 4).

#### 2.2.2. The Proliferative Activity of CACO-2 Cultured in 3D with SCIOBIO III

**Figure 5:**
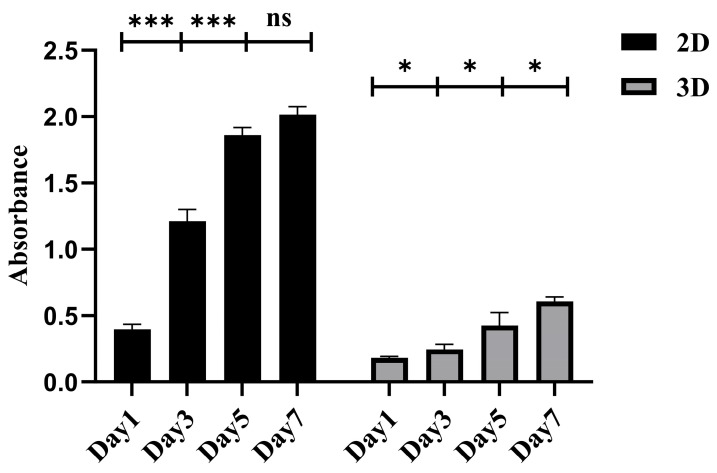
Results of proliferation activity of CACO-2 cells in 2D and SCIOBIO III short peptides matrix. The absorbance values of CACO-2 cells in 2D and 3D culture environment were detected by CCK-8 at day 1, 3, 5, and 7, respectively. * *p* < 0.05, *** *p* < 0.001.

The growth and proliferation of CACO-2 cells were monitored in both 2D and 3D cultures using the CCK-8 assay. The SCIOBIO III short peptide matrix was employed for the 3D culture. The experimental results indicated that in traditional 2D cultures without short peptides, CACO-2 cells sustained continuous growth for a week. However, starting from the fifth day, a decrease in cell proliferation activity compared to the previous period was observed. Conversely, within a 3D environment created by SCIOBIO III short peptide matrices, the cells exhibited stable and sustained proliferation throughout the 7-day observation period. This finding aligns with observations made under an optical microscope, confirming that SCIOBIO III short peptide matrices can establish a 3D microenvironment conducive to the growth and proliferation of colorectal adenocarcinoma CACO-2 cells (Figure 5).

#### 2.2.3. CACO-2 Staining of Live/Dead Cells in SCIOBIO III

This section investigates whether the SCIOBIO III short peptide matrix has potential cytotoxic effects on CACO-2 cells. The experiment was observed using live/dead cell staining. In the test reagent used in this experiment, live cells exhibit green fluorescence under a fluorescence microscope when exposed to the Calcein-AM fluorescent dye, while dead cells display red fluorescence under the same microscope when exposed to the PI fluorescent dye.

**Figure 6:**
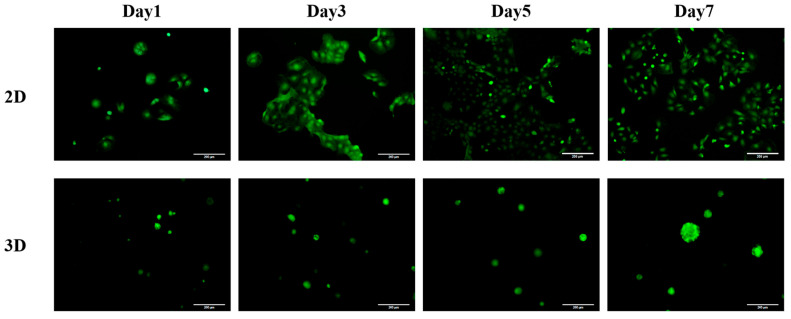
Experimental results of the cytotoxicity of CACO-2 cells in the SCIOBIO III matrix. Calcein-AM/PI staining was conducted on the 1st, 3rd, 5th, and 7th days in both 2D and 3D cultures. Live cells appeared green, while dead cells were red. Scale bar: 200 µm.

The experimental results indicate that in the conventional 2D culture system, CACO-2 cells grow in a monolayer adhering to the surface, displaying an island-like aggregation pattern with irregular shapes and sizes. In contrast, in the 3D spatial system created by the short peptide matrix SCIOBIO III, CACO-2 cells grow as spherical structures with multiple layers. Multiple cell clusters can be observed in the field of view. As the cells continue to grow, the number of cells significantly increases, and they exhibit marked enlargement. These experimental results suggest that SCIOBIO III short peptides do not display cytotoxicity and possess good biocompatibility. The SCIOBIO III short peptide matrix provides a suitable 3D culture environment for colorectal adenocarcinoma CACO-2 cells, enabling their long-term sustained growth and proliferation in vitro (Figure 6).

### 2.3. Screening of Anticancer Drugs Based on SCIOBIO III CACO-2 Cell 3D Culture

#### 2.3.1. Drug Sensitivity Detection of CACO-2 Cells Cultured in 2D

**Figure 7:**
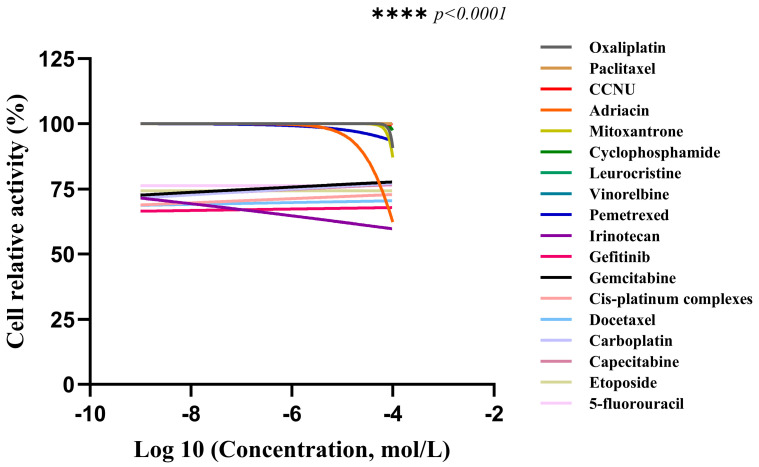
Relative activity of CACO-2 cells in colorectal adenocarcinoma after drug treatment in 2D cultures (*n* = 3), **** *p* < 0.0001.

**Figure 8:**
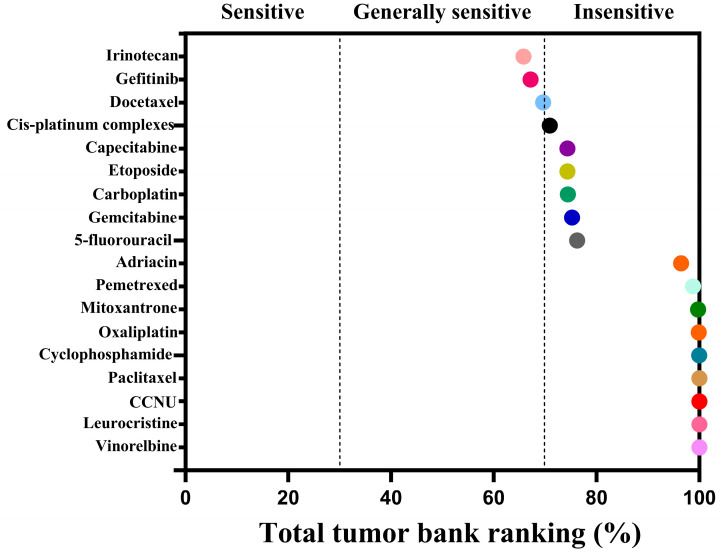
AUC curve of CACO-2 cells in colorectal adenocarcinoma after drug treatment in 2D cultures, reflecting the AUC ranking of each chemotherapeutic drug.

The cell viability of CACO-2 cells following drug treatment in a 2D culture environment was assessed using CellTiter Lumi^TM^ II 2D luminescence (Figure 7). The experimental results indicate that as the drug concentration gradient increases, the relative activity of the colorectal adenocarcinoma CACO-2 cells gradually diminishes. By employing Graphpad Prism 10 software (GraphPad Software, Inc., La Jolla, CA, USA) to calculate the AUC (Area Under the Curve) value based on the cells’ relative activity, an AUC curve can be generated (Figure 8). Ultimately, the analysis results are derived from the AUC values, which reflect the efficacy ranking and position of the chemotherapy drug within the 3D screening system for colorectal adenocarcinoma cells (Table 1). The analysis reveals a negative correlation between AUC values and drug sensitivity; in other words, lower AUC values correspond to a higher priority level of the chemotherapy drug in the total database, signifying greater sensitivity to colorectal cancer CACO-2 cells and more pronounced cytotoxic effects. In this ranking, we defined, ad hoc, the classification for AUC: an AUC value less than 35 indicates high sensitivity; values between 35 and 70 indicate general sensitivity; and values greater than 70 but less than 100 indicate low sensitivity. The drug sensitivity test results demonstrate that, based on the evaluation of overall trends, irinotecan exhibited the lowest AUC value among the 18 chemotherapy drugs, thereby ranking highest and exhibiting the greatest sensitivity to colorectal adenocarcinoma CACO-2 cells cultured in a 2D environment, with the most significant cytotoxic effects. Concurrently, gefitinib and docetaxel were ranked second and third, respectively, showing general sensitivity to CACO-2 cells. In contrast, paclitaxel, lomustine, vincristine, and vinorelbine were the least sensitive to CACO-2 cells in a 2D culture environment, exhibiting minimal killing effects.

#### 2.3.2. Drug Sensitivity Detection of CACO-2 Cells Cultured in 3D

**Figure 9:**
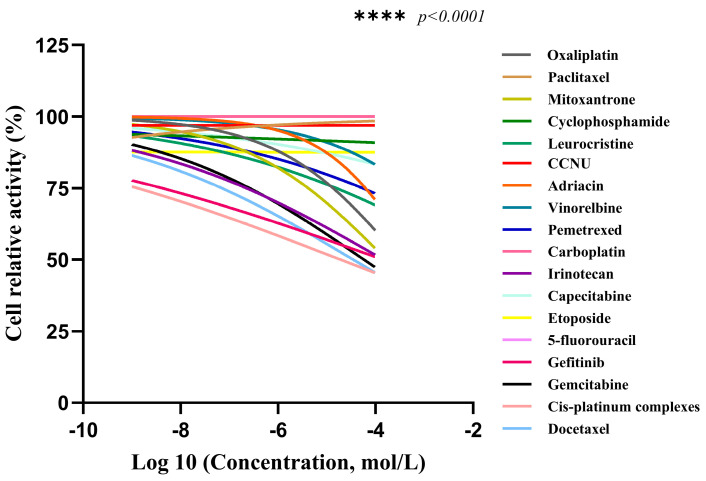
Relative activity of CACO-2 cells in colorectal adenocarcinoma after drug treatment in 3D cultures (*n* = 3), **** *p* < 0.0001.

**Figure 10:**
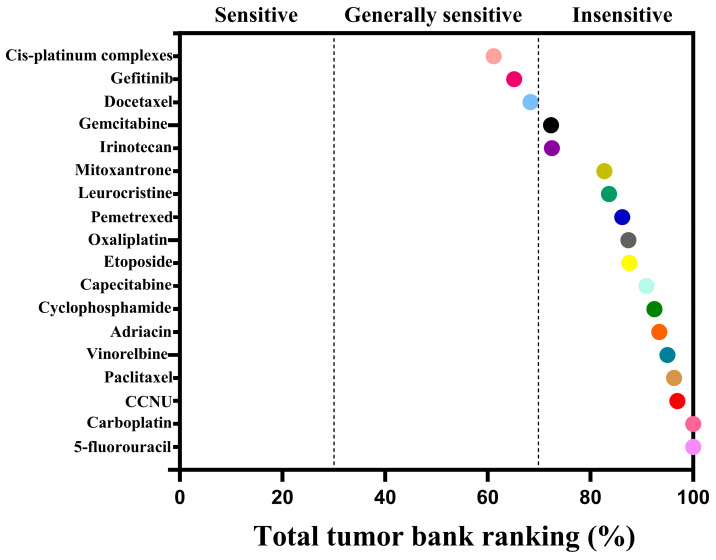
AUC curve of CACO-2 colorectal adenocarcinoma cells after drug treatment in 3D cultures, reflecting the AUC ranking of each chemotherapeutic drug.

The cell viability of CACO-2 cells following drug treatment in a 3D culture system was assessed using the CellTiter Lumi^TM^ II 3D luminescence method (Figure 9). The experimental data reveal that as the concentration of chemotherapeutic drugs increases, the survival rate of the human colorectal adenocarcinoma cell line CACO-2 exhibits a dose-dependent decrease. The AUC value for the corresponding drug can be computed based on the relative activity data using Graphpad Prism 10 software (GraphPad Software, Inc., La Jolla, CA, USA), and an AUC curve is generated (Figure 10). Ultimately, the results are analyzed according to the AUC values (the area under the curve), providing the ranking and position of the chemotherapeutic drug within the 3D screening system for colorectal adenocarcinoma cells (Table 2). The analysis indicates that there is a negative correlation between AUC values and drug sensitivity; in other words, the lower the AUC value, the higher the priority level of the chemotherapeutic drug in the total database, signifying greater sensitivity to CACO-2 cells and more pronounced cytotoxic effects. Similarly, the AUC ranking classification criteria in this section are the same as those in Section 2.3.1 above. The drug sensitivity test results demonstrate that cisplatin has the lowest AUC value among the 18 chemotherapeutic drugs, thus ranking highest and being the most sensitive to CACO-2 cells in the 3D culture system, indicating the most pronounced cytotoxic effect. Concurrently, gefitinib and docetaxel were also ranked second and third, respectively, and were generally sensitive to CACO-2 cells. However, carboplatin and 5-fluorouracil were the least sensitive to CACO-2 cells in the 3D culture system, exhibiting almost no cytotoxic effect. Meanwhile, the AUC values of 2D and 3D cultures were analyzed by *t*-test, with *p* < 0.05, showing statistical significance.

### 2.4. Discussion

In recent years, particularly with the advancement of nanotechnology, researchers have broadened their scope in the study of bioactive materials [29,30,31]. Biomaterials suitable for 3D culture must permit gas exchange with the external environment and allow for the penetration of nutrients. Therefore, these materials should possess a porous, 3D structure with appropriate pore sizes [32,33]. Within the human body, the growth of various cells is enveloped by the extracellular matrix, which contains abundant protein molecules and water [34,35].

Self-assembled short peptides are composed of natural amino acids. When the amino acid sequence varies, their corresponding physicochemical properties also change, which in turn alters their biological functions. The SCIOBIO III self-assembled short peptide, selected for this study, can assemble into a stable hydrogel matrix in the presence of PBS containing salt ions as the solvent. Its secondary structure is a stable β-sheet. Atomic force microscopy results indicate that SCIOBIO III forms interwoven nanofiber bundles, creating a scaffold structure that supports cell growth within the peptide-formed structure. Cryo-scanning electron microscopy reveals that the short peptide forms a distinct mesh-like structure, facilitating the passage of gasses and nutrients, thus providing a foundation for normal cell growth within the peptide matrix. Congo red/aniline blue staining suggests that the presence of salt ions can trigger the self-assembly of the short peptide, rapidly forming a gel. This provides a theoretical basis for subsequent 3D cell culture studies to investigate the growth and proliferation effects of colorectal adenocarcinoma cells, as well as offering a theoretical possibility for constructing 3D cultures of these cells.

Under saline conditions, self-assembled short peptides can rapidly self-assemble into permeable hydrogel scaffolds [18,26]. This process occurs very quickly when stimulated by ions. Moreover, due to the extremely high water content and the formation of the fiber scaffold, there is ample space between these fiber networks, creating an environment conducive to cell growth, proliferation, and differentiation [23,36]. During 3D culture, the biomechanical properties of self-assembled short peptide scaffolds can provide a suitable 3D spatial structure to encapsulate and fix cells in appropriate positions [37]. Moreover, they exhibit excellent permeability, allowing nutrients, growth factors, oxygen, and other substances to pass through, thereby promoting cell growth and proliferation [38]. Currently, self-assembled short peptides have been applied to the 3D culture of various types of cells [39].

The experimental results of the first part indicate that SCIOBIO III short peptides can form stable structures capable of encapsulating cells, offering a theoretical foundation for the construction of 3D cell culture systems. In the second section, SCIOBIO III was utilized to conduct in vitro 3D cultivation of colorectal adenocarcinoma CACO-2 cells, simulating the growth environment within the body. This was compared with traditional 2D cultivation. CACO-2 cells grown in the 3D system exhibited spherical growth, distributed across multiple layers of spatial structure, and remained in a state of growth and proliferation until day 7. In contrast, CACO-2 cells grown in a 2D environment showed signs of growth inhibition by day 5. The CCK-8 assay results suggest that CACO-2 cells can continue to grow in the short peptide matrix, with their proliferative activity showing a continuous upward trend; whereas in 2D cultures, although the cells proliferate, their activity decreases over time. The explanation indicates that in 2D cultures, cells experience spatial constraints leading to contact inhibition and consequently slower proliferation. However, in the 3D culture environment, the short peptides mimic the extracellular matrix, allowing cells to grow within a three-dimensional space, thereby enabling sustained growth and proliferation. This demonstrates the rationale for adopting 3D technology in cell culture. During the live/dead cell staining experiment, it was observed in 3D cultures, as cultivation time increased, CACO-2 cells grew larger and more numerous, maintaining a good survival status. This implies that SCIOBIO III short peptides have no cytotoxic effects and can effectively bind to cells. The aforementioned experimental results collectively demonstrate that the 3D culture system constructed using the SCIOBIO III short peptide matrix selected in this study can support the proliferation and growth of cells, providing an experimental basis for subsequent drug sensitivity detection through the 3D system constructed by the short peptide matrix.

In the third section, we successfully constructed a 3D culture system using colorectal adenocarcinoma CACO-2 cells as the research objects to test the sensitivity of anticancer chemotherapy drugs. The aim was to explore the feasibility of using this system for personalized and precise drug screening as a disease model. The experimental results indicate that cisplatin exhibits the highest AUC ranking in the 3D culture system, signifying its most pronounced cytotoxic effect on CACO-2 cells derived from colorectal adenocarcinoma. These findings indicate that the 3D culture system of CACO-2 cells, constructed using the SCIOBIO III short peptide matrix, is suitable for the screening of chemotherapy drugs. However, while promising, this model needs validation with primary or organoid cultures to be truly personalized. This still provides an experimental foundation for future feasibility studies, which will investigate the use of human-derived colorectal cancer organoids, also constructed with the SCIOBIO III short peptide matrix, as disease models for personalized chemotherapy drug screening. Moreover, in comparing drug screening results between 2D and 3D cultures, we observed that CACO-2 cells were most sensitive to irinotecan in a 2D environment, yet they were largely unaffected by paclitaxel, lomustine, vincristine, and vinorelbine. In contrast, in a 3D environment, CACO-2 cells were most sensitive to cisplatin, while the same drugs, paclitaxel and lomustine, had no effect on the cells in the 3D system. Additionally, gefitinib and docetaxel exhibited significant cytotoxic effects on colorectal adenocarcinoma cells in both 2D and 3D cultures. These outcomes suggest that there are both notable consistencies and disparities in drug screening between the two culture systems. We speculate that these differences may be related to the following factors. In 2D cultures, cells typically grow on a flat surface, lacking cell–cell interactions and extracellular matrix support, whereas 3D culture better mimics the in vivo environment, allowing cells to grow in a 3D space and form more complex cell–cell and cell–matrix interactions, thereby influencing drug sensitivity [40]. In 3D cultures, cell morphology and arrangement more closely resemble the in vivo state, which may lead to different drug uptake and mechanisms of action; simultaneously, cells may exhibit distinct metabolic characteristics, and these changes can affect cellular responses to drugs [41]. This study has merely investigated the feasibility of establishing a 3D culture system for colorectal adenocarcinoma cells and employing this model for drug screening, without delving into the underlying mechanisms. It is anticipated that subsequent research will conduct more comprehensive and in-depth investigations into the consistencies and differences in drug screening.

## 3. Conclusions

In conclusion, the SAP SCIOBIO III rapidly self-assembles upon ion-triggering to form a stable porous nanofiber network structure, which is conducive to the growth of colorectal adenocarcinoma cells CACO-2. It exhibits exceptional biocompatibility and mimics the extracellular matrix, enabling the development of 3D cell culture models. Furthermore, SCIOBIO III has successfully established a 3D culture model of CACO-2 and successfully conducted screening of anticancer drugs in drug sensitivity testing. The results demonstrate that the 3D culture system constructed with SCIOBIO III short peptides provides an optimized growth environment for colorectal adenocarcinoma cells in vitro. While this study has demonstrated the feasibility of drug screening in the 3D culture system, a comparative analysis of anticancer drugs identified in 2D versus 3D cultures remains critical. Future research will explore these differences to further validate the utility of this 3D platform for preclinical drug development.

## 4. Materials and Methods

### 4.1. SCIOBIO III Preparation

The sequence of SCIOBIO III is Ala-Asp-Ala-Lys-Val-Glu-Leu-Arg-Ala-Asp-Ala-Lys-Cys-Glu-Leu-Arg-NH2. All amino acids are D-form amino acids, donated by Sciobio Biotechnology Co., Ltd. (Chengdu, China).

Measure out 10 mg of the short peptide lyophilized powder and centrifuge to ensure that all residual powder adhering to the stopper falls into the bottom of the bottle (4000 rpm, 10 s). Add an equal volume of sterile distilled water to the lyophilized powder bottle using a 1 mL sterile syringe. Ultrasonicate to fully dissolve the solute, resulting in a 10 mg/mL SCIOBIO III standard storage solution. Store this solution at 4 °C for future use.

### 4.2. AFM

The fibrous network structure formed by SCIOBIO III short peptides was detected using AFM (Bruker Dimension lcon, Karlsruhe, Germany). The short peptide mother solution was diluted to a concentration of 5 mg/mL with an equal volume of phosphate buffer (PBS, Phosphate-Buffered Saline). Upon reaching room temperature equilibrium, take 10 µL of the sample and deposit it onto a mica sheet without any pretreatment. After air-drying, select the contact mode for scanning and imaging.

### 4.3. Cryo-SEM

The fibrous network structure formed by SCIOBIO III short peptides was detected using Cryo-SEM (Thermo Fisher Helios G4 UC, Hillsboro, OR, USA). To prepare a short peptide solution sample at a concentration of 5 mg/mL, take 500 µL of the short peptide mother solution at a concentration of 10 mg/mL and add 500 µL of PBS. Mix 1 mL of the liquid sample, perform cryo-fixation (rapid freezing in liquid nitrogen for 30 s), followed by gold sputter coating on the sample surface with a thickness of 5–10 nm, and finally, imaging scanning. Specific imaging parameters: the accelerating voltage was 5.00 kV; the working distance was 4.00 mm; and the magnification range was 8000–40,000×.

### 4.4. Congo Red/Aniline Blue Staining

A portion of the short peptide mother solution was diluted to 2 mg/mL with PBS, while another portion was diluted to the same concentration to 2 mg/mL using sterile double-distilled water. Ten microliters from each solution were dropped onto a slide and mixed uniformly with Congo red and aniline blue stain solutions. The samples were immediately observed under an optical microscope to capture their microscopic morphology and images, which were recorded as the initial time point (0 h). Subsequently, the remaining short peptide solution was left undisturbed at 25 °C for 24 h, after which the same staining and microscopic imaging procedures were repeated to obtain the 24 h experimental data.

### 4.5. Two-Dimensional Culture of CACO-2 Cells

Remove the CACO-2 cell tube obtained from ATCC (American Type Culture Collection) from liquid nitrogen and immediately place it in a 37 °C water bath. Shake gently until thawed, then transfer to a centrifuge tube and add 1 mL of pre-warmed medium. After mixing and centrifugation (1000 rpm, 5 min), resuspend the pellet with 2 mL of medium. Seed the cells in a T25 flask containing 5 mL of medium and culture (37 °C, 5% CO_2_) with an initial cell count of approximately 800,000. When cell confluence reaches 80%, passage the cells to a T75 flask. Once the adherent cells reach 80–90% confluence, select CACO-2 cells in the logarithmic growth phase with intact morphology for the 3D culture.

### 4.6. Three-Dimensional Culture of CACO-2 Cells

Remove the culture flask from the incubator and discard the old medium in a biosafety cabinet. Wash the adherent cells in 3 mL of sterile PBS, repeating this process three times until the solution becomes clear, to remove the dead cells to the greatest extent possible. Aspirate PBS, add 2 mL of 0.25% trypsin solution and incubate in the incubator for 2 min. Terminate digestion when the cells appear bright and round under the microscope and resuspend in complete medium. Transfer to a centrifuge tube and centrifuge at 1000 rpm for 5 min, then resuspend in 1 mL of sterile PBS. Count cells and prepare a short peptide–PBS cell suspension at a 1:1 ratio by mixing 25 μL of cell suspension with 25 μL of 10 mg/mL SCIOBIO III, resulting in a final SCIOBIO III concentration of 5 mg/mL. Inoculate 50 μL per well into a 96-well plate, add 10 μL of complete medium, then allow to settle for 5 min. Add another 40 μL of medium, incubate for 20 min, and then add 100 μL of complete medium for continued culture (stepwise addition of medium ensures cells receive sufficient nutrients during SCIOBIO III solidification to support growth). The day after seeding is designated as Day 1 of plating, with medium changes every two days, for a total culture duration of 7 days.

### 4.7. CCK-8 Detection

The impact of SCIOBIO III short peptides on the proliferation of CACO-2 cells in 2D and 3D cultures was examined using CCK-8 and a kit.

Following the aforementioned 3D cell culture protocol, a CACO-2 cell suspension was thoroughly mixed with SCIOBIO III short peptides and seeded into a 96-well plate for 3D culture as the experimental group, with a final peptide concentration of 5 mg/mL. A 2D culture of CACO-2 cells was used as the control group. Both groups were seeded into 96-well culture plates at a density of 5 × 10^3^ cells per well, with three replicate wells per group. Cell proliferation assays were conducted on the 1st, 3rd, 5th, and 7th days post-inoculation of CACO-2 cells. Before each assessment, the culture plates were removed from the incubator, and the medium was replaced with fresh medium, without the need for pre-equilibration before detection. After adding 10 μL of CCK-8 detection reagent to each well under light-protected conditions, the plates were returned to the 37 °C incubator for an additional 2 h incubation. A multi-functional microplate reader (Bio Tek H1MFD, Winooski, VT, USA) was used to measure the optical density values at a 450 nm wavelength for each experimental and control group, and the experimental data were systematically recorded.

### 4.8. Live/Dead Cell Staining

The double staining technique for live and dead cells was employed to assess the cytotoxicity of SCIOBIO III short peptides on CACO-2 cells, aiming to demonstrate the biocompatibility of the short peptide. Calcein AM is capable of entering living cells and being hydrolyzed to produce the polar molecule Calcein, which is unable to penetrate the cell membrane, thereby remaining within the cell and emitting intense green fluorescence. In contrast, dead cells either cannot or rarely produce Calcein. Propidium iodide (PI) cannot penetrate the cell membranes of living cells and can only reach the nucleus by passing through the disordered regions of dead cell membranes, resulting in red fluorescence. Consequently, the concurrent use of Calcein AM and PI can effectively differentiate between living and dead cells.

The SCIOBIO III short peptide matrix was combined with CACO-2 cells and cultured in plates as the experimental group, with a control group consisting of CACO-2 cells in a 2D culture. The medium volume per well is 150 μL. Each well, containing 2500 cells, achieves optimal fluorescence detection performance in both 2D and 3D culture systems. Staining was conducted on the 1st, 3rd, 5th, and 7th days post-inoculation of the CACO-2 cells. After removing the culture plates, the old medium in each well was aspirated using a pipette. The 2D culture group was washed three times with sterile PBS preheated in a water bath, whereas the 3D culture experimental group was soaked in preheated sterile PBS for 5 min to remove the culture medium to the greatest extent possible, ensuring the short peptide matrix in each well remained colorless and transparent. A 100 μL Calcein-AM/PI fluorescent staining solution (Beyotime, Shanghai, China, BL130S) was added to each well, with the reagents being prepared immediately and used under light protection. After adding the reagents, the 96-well plate was placed back into the incubator for 15 min. Following staining, the culture plates were removed, washed with sterile PBS, and finally, an inverted fluorescence microscope (Thermo Fisher, Waltham, MA, USA) was used to qualitatively observe the staining results and photograph the records.

### 4.9. Drug Sensitivity Detection of CACO-2 Cells in 2D/3D Culture

#### 4.9.1. Drug Sensitivity Detection of CACO-2 Cells in 2D Cultures

Drug preparation: Drug concentration gradients from 10^−8^ to 10^−3^ mol/L were prepared using the serial dilution method. Eighteen chemotherapeutic drugs were selected based on clinical relevance and arranged in six gradients. Drugs were dissolved in solvent (DMSO or bacterial water) to prepare concentration solutions. Efficacy was evaluated by AUC, with the minimum value indicating the highest cytotoxic effect on CACO-2 cells.

Two-dimensional culture and plating of CACO-2 cells: Remove T75 flask from incubator, digest for 2 min with 0.25% trypsin, and centrifuge (1000 rpm, 5 min). Resuspend in complete medium, and count cells. Seed at 100 μL/well in 96-well plate, at 4000 cells/well. Establish the drug treatment group with three replicate wells and control groups. Return the cells to the incubator for adhesion and growth. The first day is recorded 24 h after the plate is seeded.

Two-dimensional culture with drug addition: After seeding the cells and culturing them for two days, proceed with the drug treatment. Aspirate 80 μL of old medium and add 70 μL of new medium to each well. Add 10 μL of chemotherapeutic drugs at specified concentrations. At this juncture, the final concentration gradient of the chemotherapeutic drugs in the 2D culture system spans from 10^−9^ to 10^−4^ mol/L, and this range is utilized as the *X*-axis on the result graph. Incubate the 96-well plate with drugs at 37 °C, 5% CO_2_. After 72 h, evaluate cytotoxicity on CACO-2 cells.

The CellTiter Lumi^TM^ II 2D Luminescent Cell Viability Assay Kit (Beyotime, Shanghai, China, C0056M) was used to measure ATP activity and drug sensitivity in CACO-2 cells. After the drug treatment, the plates were equilibrated at room temperature and 100 μL of detection reagent was added. The plates were shaken for 2 min and left undisturbed for 10 min (60 rpm). The chemiluminescence detection was performed using a multifunctional microplate reader, with a detection time of 1 s per well. Finally, based on the obtained experimental data, the relative viability of the cells was calculated and the AUC curve result graph was plotted.

#### 4.9.2. Drug Sensitivity Detection of CACO-2 Cells in 3D Cultures

The preparation method for the drug is consistent with that of the above 2D culture.

Three-dimensional culture and seeding of CACO-2 cells: Mix peptides with PBS cell suspension at a 1:1 ratio and seed into 96-well plates at 100 μL per well. Ensure that there are 10,000 cells per well. Establish the drug treatment group with three replicate wells and control groups. Allow cells to settle for 20 min, add 100 μL complete medium, and continue growth. The first day is recorded 24 h after the plate is seeded.

Three-dimensional culture drug addition: After seeding the cells and culturing them for two days, proceed with the drug treatment. Aspirate 80 μL of old medium and add 70 μL of new medium to each well. Add 10 μL of chemotherapeutic drug at the correct gradient. At this juncture, the final concentration gradient of the chemotherapeutic drugs in the 3D culture system spans from 10^−9^ to 10^−4^ mol/L, and this range is utilized as the *X*-axis on the result graph. Incubate the 96-well plate with drugs at 37 °C, 5% CO_2_. After 72 h, evaluate the cytotoxicity on CACO-2 cells.

The CellTiter Lumi^TM^ II 3D Luminescent Cell Viability Assay Kit (Beyotime, Shanghai, China, C0062M) was used to measure ATP cell activity and test drug sensitivity in CACO-2 cells. After the drug treatment, the plates were equilibrated at room temperature for 10 min, then 100 μL of detection reagent was added and the plates were shaken for 5 min (60 rpm). The plates were left undisturbed for 25 min before the chemiluminescence detection was performed using a multifunctional microplate reader, with a detection time of 1 s per well. Cell viability was calculated and AUC curves were plotted based on the data.

### 4.10. Data Analysis

All experimental data in this study were analyzed and processed using GraphPad Prism 10.0 (GraphPad Software, Inc., La Jolla, CA, USA). The *t*-test was used to assess comparisons between the two groups. One-way ANOVA was used to evaluate comparisons among three or more mutually independent groups. *p* < 0.05 was considered statistically significant.

## 5. Limitations

This study utilized SCIOBIO III to establish a 3D culture system for CACO-2 cells and conducted anticancer drug sensitivity testing through this system to achieve the purpose of drug screening. However, certain limitations in the study may affect its generalizability. Firstly, although 3D cultures can better simulate the actual tumor microenvironment, they may still not fully reflect the complex in vivo interactions, such as the presence of immune cells and dynamic changes in the tumor microenvironment. Secondly, while both 2D and 3D cultures can be used for drug sensitivity testing in vitro, the results still need to be combined with clinical practice. Finally, the growth stage of cells in culture may influence their sensitivity to drugs, and the lack of comprehensive evaluation across different growth stages could impact the generalizability of the results.

## Figures and Tables

**Table 1:** Chemotherapy drugs sensitivity of colorectal adenocarcinoma CACO-2 cells in 2D cultures.

Drug Classification	Name of the Drug	AUC	Sensibility
Chemotherapy drugs	Irinotecan	65.78	Generally sensitive
	Gefitinib	67.14	Generally sensitive
	Docetaxel	69.60	Generally sensitive
	Cis-platinum Complexes	70.88	insensitivity
	Capecitabine	74.32	insensitivity
	Etoposide	74.34	insensitivity
	Carboplatin	74.42	insensitivity
	Gemcitabine	75.22	insensitivity
	5-fluorouracil	76.24	insensitivity
	Adriacin	96.46	insensitivity
	Pemetrexed	98.78	insensitivity
	Mitoxantrone	99.76	insensitivity
	Oxaliplatin	99.90	insensitivity
	Cyclophosphamide	99.96	insensitivity
	Paclitaxel	100.00	insensitivity
	CCNU	100.00	insensitivity
	Leurocristine	100.00	insensitivity
	Vinorelbine	100.00	insensitivity

**Table 2:** Chemotherapy drugs sensitivity of colorectal adenocarcinoma CACO-2 cells in 3D cultures.

Drug Classification	Name of the Drug	AUC	Sensibility
Chemotherapy drugs	Cis-platinum Complexes	61.16	Generally sensitive
	Gefitinib	65.14	Generally sensitive
	Docetaxel	68.34	Generally sensitive
	Gemcitabine	72.30	insensitivity
	Irinotecan	72.50	insensitivity
	Mitoxantrone	82.70	insensitivity
	Leurocristine	83.60	insensitivity
	Pemetrexed	86.18	insensitivity
	Oxaliplatin	87.40	insensitivity
	Etoposide	87.58	insensitivity
	Capecitabine	90.92	insensitivity
	Cyclophosphamide	92.44	insensitivity
	Adriacin	93.42	insensitivity
	Vinorelbine	95.00	insensitivity
	Paclitaxel	96.28	insensitivity
	CCNU	96.92	insensitivity
	Carboplatin	100.00	insensitivity
	5-fluorouracil	100.00	insensitivity

## Data Availability

The data presented in this study are available in the article.
